# Xueshuantong Injection in Treating Deep Venous Thrombosis: A Systematic Review and Trial Sequential Analysis

**DOI:** 10.1155/2021/6622925

**Published:** 2021-04-07

**Authors:** Wenhui Li, Feng Xu, Renyan Huang, Weijing Fan, Changgeng Fu, Lei Xu, Xvhong Wang, Huimin Lu, Yuanxiang Li

**Affiliations:** ^1^Shanghai University of Medicine & Health Sciences, Shanghai, China; ^2^Shuguang Hospital Affiliated to Shanghai University of Traditional Chinese Medicine, Shanghai, China; ^3^Dongfang Hospital Affiliated to Beijing University of Chinese Medicine, Beijing, China

## Abstract

**Aims:**

In recent years, the incidence of deep venous thrombosis (DVT) presents an increasing trend year by year. The current evidence regarding the efficacy and safety of Xueshuantong injection for DVT is controversial. This systematic review (SR) aimed to assess the efficacy and safety of Xueshuantong injection in the treatment of DVT systematically and provide an evidence-based reference for clinical treatment.

**Methods:**

Nine electronic databases were used to identify the literature consisting of randomized controlled trials (RCTs) with a date of search of 1 November 2020. Clinical effective rate and incidence rate of adverse events were investigated as primary outcomes. Patency rate of femoral vein, patency rate of popliteal vein, patency rate of posterior tibial vein, circumference difference, activated partial thromboplastin time (APTT), and D-dimer (D-D) were investigated as secondary outcomes. Revman 5.4.1 was used to analyze the results. Analysis of the power of evidence was performed with Trial Sequential Analysis (TSA).

**Results:**

A total of 12 articles including 1018 patients were included. The results of the meta-analysis showed that the clinical effective rate in the experimental group was higher than that in the control group, the incidence rate of adverse events in the experimental group was higher than that in the control group; after the operation, the patency rate of femoral vein, patency rate of popliteal vein, patency rate of posterior tibial vein, circumference difference, APTT, and D-D in the experimental group were significantly improved compared with those in the control group, and the difference between the groups was statistically significant. TSA suggested that the meta-analysis concerning the clinical effectiveness of Xueshuantong injection in the treatment of DVT was of adequate power to reach firm conclusions.

**Conclusion:**

Based on the current analysis, Xueshuantong injection as an add-on treatment provided better treatment effect for DVT with adequate power but this benefit should be considered with caution because of the small number of studies included in the meta-analysis and the high or unclear risk of bias of the included trials, suggesting that further studies are needed.

## 1. Introduction

Deep venous thrombosis (DVT) is a common critical disease with very high incidence in elderly inpatients. Studies have shown that almost 20% of patients scheduled for orthopedic surgery have DVT, despite the use of preventative strategies [1, 2]. In addition, if patients with DVT do not receive timely and effective treatment, almost 50% will develop DVT syndrome, significantly affecting their work and life, and 6% will die of pulmonary embolism [3, 4]. Some scholars of traditional Chinese medicine consider the pathogenesis of DVT to be related to disorders of Qi and blood, and that the first line of treatment involves the promotion of blood flow. Xueshuantong injection is a safe and effective traditional Chinese medicine which is widely used in the treatment of various thrombus-related diseases including DVT [5]. While clinical studies in recent years have shown that Xueshuantong injection can treat DVT effectively, reducing symptoms with few adverse events, its effectiveness and safety have not been comprehensively and systematically evaluated.

This study systematically evaluated the efficacy and safety of Xueshuantong injection in treating DVT, in order to provide an evidence-based medical basis for the treatment of DVT.

## 2. Materials and Methods

The protocol for this systematic review has been developed according to the Preferred Reporting Items for Systematic Reviews and Meta-Analyses (PRISMA) guidelines [6].

It has been also registered on the INPLASY database (registration number: INPLASY2020120117).

### 2.1. Search Strategy

The Chinese databases include Chinese National Knowledge Infrastructure Database (CNKI); the Chongqing VIP Database; Chinese Biomedical medical Database (CBM); and Wanfang Database.

The English databases include PubMed, Cochrane Library, Web of Science, Embase, and MEDLINE.

The search terms are “Panax notoginseng saponins”; “Panaxnotoginseng extract”; “Xueshuantong”; “XUESAITONG injection”; “Deep Venous Thrombosis”; “DVT”; “efficacy”; and “safety”.

The search time is from the establishment time of each database to November 1, 2020.

The complete search strategy for PubMed is reported in Table 1. The search strategy for PubMed will be adapted for use in other databases.

Add search is the search by mainstream search engines (Baidu, Google); Clinical Research Registry (WHO International Clinical trial Registration platform; China Clinical trial Registration Center).

At the same time, the two researchers searched the literature independently, cross-checked the results, and further searched the references according to the search results.

### 2.2. Inclusion Criteria

#### 2.2.1. Research Type

The type is randomized controlled trials (RCTs).

#### 2.2.2. Study Population

The original study clearly pointed out that the included patients were in line with the relevant diagnosis of DVT. Refer to the guidelines for diagnosis and treatment of deep vein thrombosis [7] (according to the symptoms, clinical signs, blood coagulation tests, color Doppler ultrasound results, etc).

#### 2.2.3. Intervention

The experimental group received Xueshuantong injection or Xueshuantong combined with conventional treatment (anticoagulation, thrombolysis, pressure treatment, and other clinical guidelines recommended treatments).

### 2.3. Control Group: Conventional Treatment or Placebo

#### 2.3.1. Outcomes

*(1) Primary Outcomes*. Clinical effective rate (the circumference difference of the lower limbs was less than 2.5 cm). Refer to the standard for diagnosis and efficacy of TCM Internal Medicine [8].

Incidence rate of adverse events:

*(2) Secondary Outcomes*.

  Patency rate of femoral vein
  Patency rate of popliteal vein
  Patency rate of posterior tibial vein
  Circumference difference
  Activated partial thromboplastin time (APTT)
  The D-dimer (D-D)

### 2.4. Exclusion Criteria

The exclusion criteria were as follows: nonoriginal research; the outcome indicators not matching; key research data not fully displayed and not being able to be obtained; research reports repeatedly published; and data errors.

### 2.5. Data Extraction

The two researchers independently screened the included literature and extracted the data. The differences encountered in the process can be resolved through negotiation, or they can consult experts in the research team to record the process.

### 2.6. Dealing with Missing Data

In the case of missing data, we contacted the original trial investigators to request missing data whenever possible.

### 2.7. Literature Quality Evaluation

The quality evaluation tool is Bias Risk Assessment Tool developed by Cochrane Collaboration Network.

Evaluation items are as follows: how random sequences are generated; whether distribution is hidden; whether researchers and subjects are blinded; whether study outcomes are blinded; whether outcome data remain intact; selective reporting; and whether there are other biases.

### 2.8. Statistical Analysis

RevMan 5.4.1 was used for data analysis and odds ratio (OR) or relative risk (RR) was used for the dichotomous variable and mean difference (MD) or standardized mean difference (SMD) was used for continuous variables and 95% CI was used to test the effect.

The heterogeneity was analyzed by using the *Q* value test and *I*^2^ test. If *P* > 0.1 and *I*^2^ ≤50%, no heterogeneity was judged, and the fixed effect model was used for meta-analysis. *P* > 0.1, *I*^2^ >50% indicate heterogeneity; random effect model is used for meta-analysis; and the source of heterogeneity is found through sensitivity analysis or subgroup analysis (a sufficient number of studies are included). If the heterogeneity cannot be solved, the meta-analysis will be abandoned and descriptive analysis will be used.

### 2.9. Trial Sequential Analysis

Trial Sequential Analysis (TSA) 0.9.5.10 (https://www.ctu.dk/tsa/) was performed to calculate the required information size (RIS), alpha spending function, trial sequential monitoring boundaries for benefits and harms, and futility boundaries assessment.

## 3. Results

### 3.1. Results of the Search

**Figure 1:**
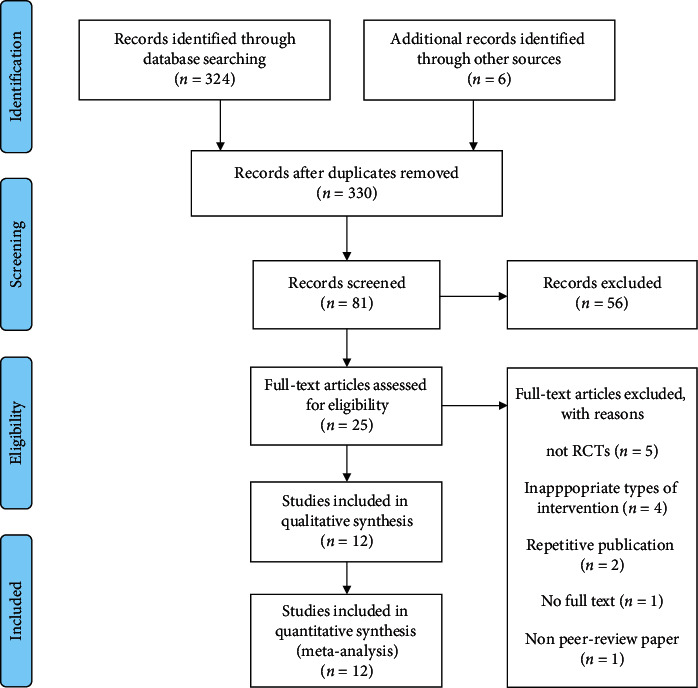
Flow chart of the search strategy.

A total of 330 potentially corresponding studies were identified by our primary search. After removing duplicate records, we identified 25 studies from 330 studies for review of the full text. A total of 13 studies were exempted for the following reasons: 5 articles are not RCTs, 4 articles are inappropriate types of intervention, 4 articles are repetitive publication, 1 article has not full text and 1 article is not peer-review paper. Of the 12 included studies, 1018 patients were used in this meta-analysis, as is shown in Figure 1.

### 3.2. Basic Characteristics of Included Studies

The basic characteristics of the 12 RCTs are summarized in Table 2.

### 3.3. Assessment of Study Quality and Risk of Bias

**Figure 2:**
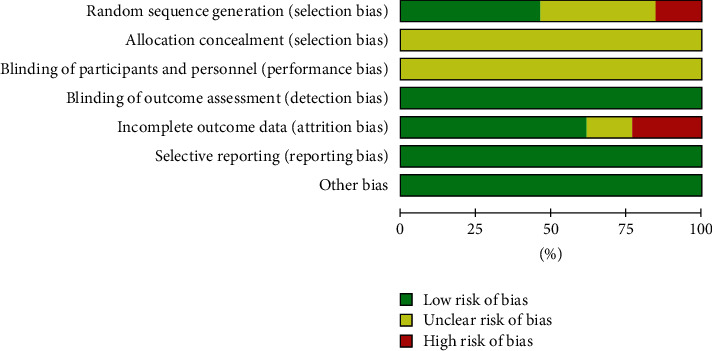
Risk of bias graph with overall percentages of bias for each domain.

**Figure 3:**
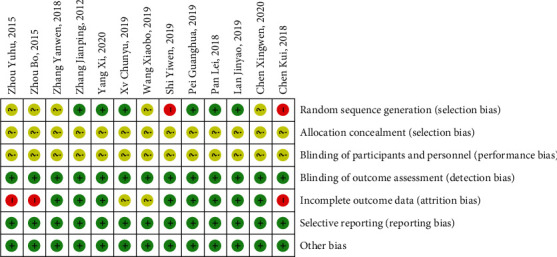
Risk of bias summary across all included studies.

Of the 12 studies, 8 [9–12, 14–16, 20] reported random method, 6 studies [9–11, 14, 16, 20] used the random number table to generate random numbers; 2 studies [12, 15] generated random numbers with a high risk of bias; and none of the studies reported allocation concealment. Figures 2 and 3 show the risk of bias summary of the included studies.

### 3.4. Statistical Analysis

#### 3.4.1. Clinical Effective Rate

**Figure 4:**
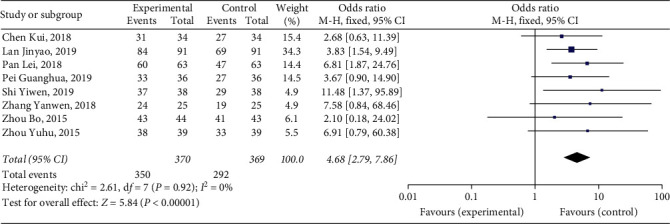
Forest plot of clinical effective rate for Xueshuantong injection in treating DVT.

Eight studies [10–12, 15–19] reported clinical effective rate, with a total of 739 patients. The results showed that Xueshuantong injection was superior to conventional treatment in the improvement of clinical effective rate in the treatment of DVT, and the difference was statistically significant (OR = 4.68, 95% CI [2.79, 7.86], *P* < 0.00001). Considerably, there was no heterogeneity among the studies (*P*=0.92, *I*^2^ = 0%), and a fixed effect model was used for meta-analysis, the results of which are shown in Figure 4.

#### 3.4.2. Incidence Rate of Adverse Events

**Figure 5:**
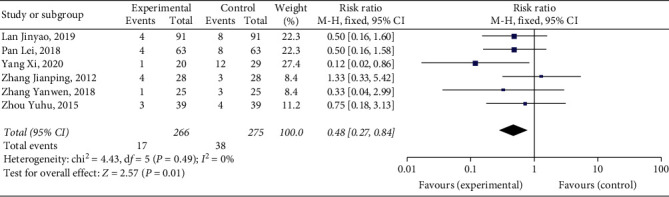
Forest plot of the incidence rate of adverse events for Xueshuantong injection in treating DVT.

Six studies [9, 10, 16, 17, 19, 20] reported the incidence rate of adverse reactions, with a total of 541 patients. No serious adverse reactions occurred in all the studies. The results showed that Xueshuantong injection was superior to conventional treatment in the improvement of the incidence rate of adverse events in the treatment of DVT, and the difference was statistically significant (OR = 0.48, 95% CI [0.27, 0.84], *P*=0.01). Considerably, there was no heterogeneity among the studies (*P*=0.49, *I*^2^ = 0%), and a fixed effect model was used for meta-analysis, the results of which are shown in Figure 5.

#### 3.4.3. Patency Rate of Femoral Vein

**Figure 6:**
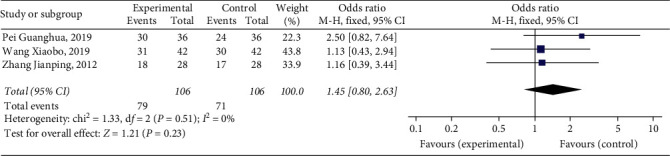
Forest plot of patency rate of femoral vein for Xueshuantong injection in treating DVT.

Three studies [11, 13, 20] reported patency rates of femoral vein, with a total of 212 patients. The results showed that Xueshuantong injection was superior to conventional treatment in the improvement of patency rates of the femoral vein in the treatment of DVT, and the difference was statistically significant (OR = 1.45, 95% CI [0.80, 2.63], *P*=0.23). Considerably, there was no heterogeneity among the studies (*P*=0.51, *I*^2^ = 0%), and a fixed effect model was used for meta-analysis, the results of which are shown in Figure 6.

#### 3.4.4. Patency Rate of Popliteal Vein

**Figure 7:**
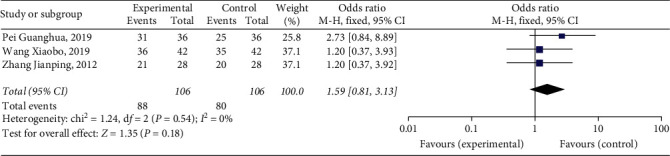
Forest plot of patency rate of popliteal vein for Xueshuantong injection in treating DVT.

Three studies [11, 13, 20] reported patency rates of popliteal veins, with a total of 212 patients. The results showed that Xueshuantong injection was superior to conventional treatment in the improvement of patency rates of popliteal veins in the treatment of DVT, and the difference was statistically significant (OR = 1.59, 95% CI [0.81, 3.13], *P*=0.18). Considerably, there was no heterogeneity among the studies (*P*=0.54, *I*^2^ = 0%), and a fixed effect model was used for meta-analysis, the results of which are shown in Figure 7.

#### 3.4.5. Patency Rate of Posterior Tibial Vein

**Figure 8:**
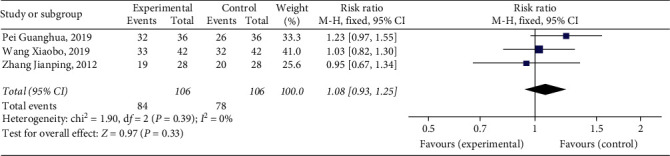
Forest plot of patency rate of posterior tibial vein for Xueshuantong injection in treating DVT.

Three studies [11, 13, 20] reported patency rate of the posterior tibial vein, with a total of 212 patients. The results showed that Xueshuantong injection was superior to conventional treatment in the improvement of patency rate of the posterior tibial vein in the treatment of DVT, and the difference was statistically significant (OR = 1.08, 95% CI [0.93, 1.25], *P*=0.33). Considerably, there was no heterogeneity among the studies (*P*=0.39, *I*^2^ = 0%), and a fixed effect model was used for meta-analysis, the results of which are shown in Figure 8.

#### 3.4.6. Circumference Difference of Upper Knee Limb

**Figure 9:**
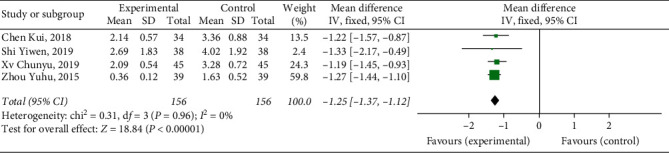
Forest plot of patency rate of circumference difference of upper knee limb for Xueshuantong injection in treating DVT.

Four studies [12, 14, 15, 19] reported a circumference difference of upper knee limb, with a total of 312 patients. The results showed that Xueshuantong injection was superior to conventional treatment in the improvement of circumference difference of upper knee limb in the treatment of DVT, and the difference was statistically significant (OR = −1.25, 95% CI [−1.37, −1.12], *P* < 0.00001). Considerably, there was no heterogeneity among the studies (*P*=0.96, *I*^2^ = 0%), and a fixed effect model was used for meta-analysis, the results of which are shown in Figure 9.

#### 3.4.7. Circumference Difference of Lower Knee Limb

**Figure 10:**
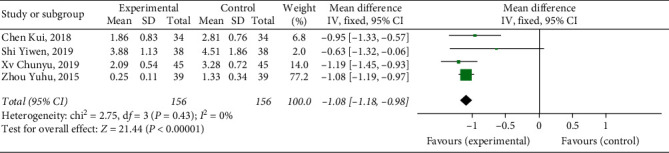
Forest plot of patency rate of circumference difference of lower knee limb for Xueshuantong injection in treating DVT.

Four studies [12, 14, 15, 19] reported circumference difference of lower knee limb, with a total of 312 patients. The results showed that Xueshuantong injection was superior to conventional treatment in the improvement of Circumference difference of lower knee limb in the treatment of DVT, and the difference was statistically significant (OR = −1.08, 95% CI [−1.18, −0.98], *P* < 0.00001). Considerably, there was no heterogeneity among the studies (*P*=0.43, *I*^2^ = 0%), and a fixed effect model was used for meta-analysis, the results of which are shown in Figure 10.

#### 3.4.8. Activated Partial Thromboplastin Time

**Figure 11:**
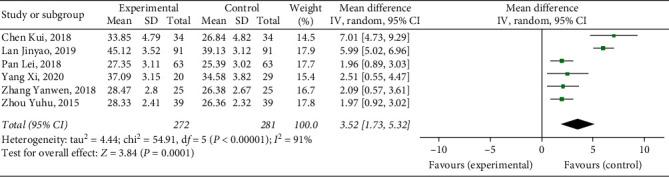
Forest plot of APTT for Xueshuantong injection in treating DVT (6 studies).

Six studies [9, 10, 15–17, 19] reported APTT, with a total of 553 patients. The results showed that Xueshuantong injection was superior to conventional treatment in the improvement of APTT in the treatment of DVT, and the difference was statistically significant (OR = 3.52, 95% CI [1.73, 5.32], *P*=0.00001). Considerable heterogeneity was found between the studies (*P* < 0.00001, *I*^2^ = 91%), and a random effects model was used for meta-analysis, the results of which are shown in Figure 11.

**Figure 12:**
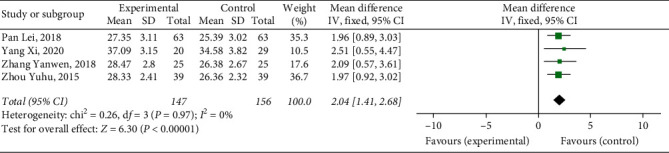
Forest plot of APTT for Xueshuantong injection in treating DVT (4 studies).

After excluding the two studies [10, 15], the heterogeneity of APTT was significantly reduced (*P*=0.97, *I*^2^ = 0%), and the two studies were judged as the source of heterogeneity, the results of which are shown in Figure 12.

#### 3.4.9. D-Dimer

**Figure 13:**
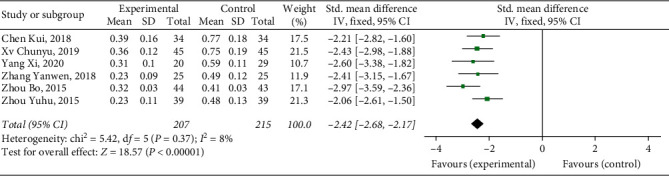
Forest plot of D-D for Xueshuantong injection in treating DVT.

Six studies [9, 14, 15, 17–19] reported D-D, with a total of 422 patients. The results showed that Xueshuantong injection was superior to conventional treatment in the improvement of D-D in the treatment of DVT, and the difference was statistically significant (OR = −2.42, 95% CI [−2.68, −2.17], *P* < 0.00001). Considerable heterogeneity was found between the studies (*P*=0.37, *I*^2^ = 8%), and a fixed effect model was used for meta-analysis, the results of which are shown in Figure 13.

### 3.5. Publication Bias

A maximum of 8 outcome indices were available so no assessment of publication bias (e.g., funnel plot) could be made and the possibility of this form of bias cannot be excluded.

### 3.6. Sensitivity Analysis

Sensitivity analysis was carried out by changing the statistical model and removing the recombination effect of a large sample study. This process did not change the results, indicating that the results of this study were robust.

### 3.7. Trial Sequential Analysis

**Figure 14:**
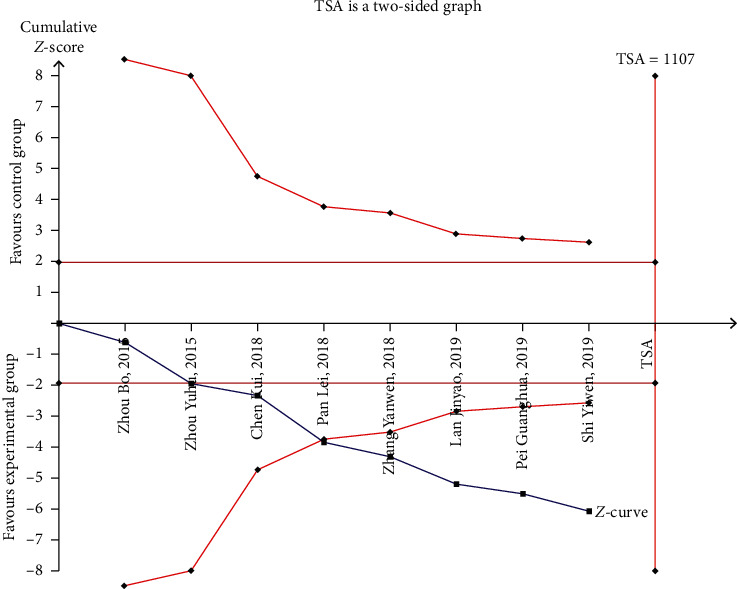
Trial Sequential Analysis of clinical efficiency for Xueshuantong injection in treating DVT.

The type I and type II errors were set at 0.05 and 0.2, respectively. The sample size was calculated with statistical power at 80%. The clinical effectiveness of Xueshuantong injection in the treatment of DVT was analyzed by experimental sequence analysis. The value (curve) crossed the traditional boundary value after the second study and crossed the TSA boundary value after the fourth study, indicating that the positive conclusion had been obtained before the amount of sample information in the current cumulative study had not reached the expected amount of information; that is, Xueshuantong injection is more effective than conventional treatment in the treatment of DVT, and that no further studies were needed for verification, as shown in Figure 14.

## 4. Discussion

Deep venous thrombosis refers to abnormal blood flow and coagulation in the deep venous system [21]. Clinical patients have lower limb swelling, local skin temperature rise, and pain, and may suffer fatal pulmonary embolism in severe cases [21–24]. In traditional Chinese medicine, this condition is referred to as “thigh swelling” and treatment is based on promoting blood circulation, removing blood stasis, and replenishing qi. Xueshuantong injection is a traditional Chinese medicine injection made from total saponins extracted from Panax notoginseng, a modern form of Chinese patent medicine [25, 26]. The main components of Xueshuantong injection are ginseng and Panax notoginseng. Panax notoginseng has the effect of dispersing blood stasis and stopping bleeding and pain and detumescence. Panax notoginseng can reduce the levels of CD62p, CD63, GPIIb/IIIa, FIB, and D-dimer in plasma of patients with deep venous thrombosis and thus play a therapeutic role. Ginseng preparation can also improve blood hypercoagulable state [27]. In addition, some studies have shown that Xueshuantong injection may have an antithrombotic effect by inhibiting platelet aggregation, improving blood flow, and maintaining an environment conducive to normal blood flow [28–30].

The results of this study show that Xueshuantong injection can treat deep venous thrombosis effectively, with fewer adverse reactions and significantly better outcomes (including deep vein patency, circumference of lower extremities, D-dimer, and APTT) than conventional treatment. The results of the sequential analysis showed that the results of this meta-analysis are robust and may therefore have implications for evidence-based clinical practice. However, in view of the small sample size included and the low quality of literature methodology, the strength of this finding is limited, and more high-quality studies are needed to improve the level of evidence in the future.

Limitations of this study: this study compares Xueshuantong injection with conventional treatment, the latter encompassing anticoagulation, thrombolysis, and physiotherapy, which may have introduced heterogeneity. In addition, the included studies were all sourced from Chinese research databases. No relevant research was found in English language databases, and the representation of only one sector of the global population may have introduced bias. In the process of literature screening in this study, several articles were excluded because key data could not be obtained by the author, which may have biased the results. In addition, the quality of the research included in this study was low, with small sample sizes in some cases, which further reduces the certainty of conclusions.

Future prospects: future clinical research should use homogeneous outcome indicators to gradually establish a core outcome index set with indicators related directly to patient benefits. Higher quality research is needed with high integrity, standardized reporting, and reduced bias.

## 5. Conclusion

Based on the current analysis, compared with control, Xueshuantong injection as an add-on treatment provided better clinical efficiency for DVT with adequate power, while with fewer adverse reactions and significantly better outcomes (including patency of deep vein, circumference of lower extremities, D-D, and APTT), but this benefit should be considered with caution because of the small number of studies included in the meta-analysis and the high risk of bias of the included trials, suggesting that further studies are needed.

## Figures and Tables

**Table 1:** Search strategy for PubMed.

Search strategy (PubMed database)	
Number	Search terms
#1	Xue Shuan Tong (MeSH)
#2	xueshuantong
#3	Xueshuantong injection
#4	Panax notoginseng extract
#5	Panax notoginseng
#6	#1 OR #2 OR #3 OR #4 OR #5
#7	Deep venous thrombosis (MeSH)
#8	DVT
#9	venous thrombosis
#10	Deep venous thrombosis
#11	thrombosis
#12	#7 OR #8 OR #9 OR #10 OR #11
#13	Randomized controlled trial (MeSH)
#14	Randomized controlled trial
#15	Controlled clinical trial
#16	Clinical trial
#17	trial
#18	#13 OR #14 OR #15 OR #16 OR #17
#19	#6 and #12 and #18

**Table 2:** Characteristics of the included studies.

Included study	Sample size (T/C)	Treatment		Gender (M/F)		Age (years)		Treatment course (days)	Random method	Evaluation indicators
		Experimental group	Control group	Experimental group	Control group	Experimental group	Control group			
Yang Xi, 2020 [9]	20/29	Xueshuantong injection + low molecular weight heparin	Low molecular weight heparin	0/20	0/29	56.45 ± 19.74	57.05 ± 21.74	14	Random number table	7；8；9
Lan Jinyao, 2019 [10]	91/91	Xueshuantong injection + low molecular weight heparin	Low molecular weight heparin	50/41	42/49	51.72 ± 16.82	48.73 ± 18.27	14	Random number table	1；7；9
Pei Guanghua, 2019 [11]	36/36	Xueshuantong injection + urokinase injection	Urokinase injection	20/16	21/15	62.9 ± 1.1	62.8 ± 1.2	14	Random number table	1；2；3；4
Shi Yiwen, 2019 [12]	38/38	Xueshuantong injection + rivaroxaban	Rivaroxaban	45/31		57.7 ± 4.5		14	Drawing lots	1；5；6
Wang Xiaobo, 2019 [13]	42/42	Xueshuantong injection + urokinase injection	Urokinase injection	27/15	25/17	68.7 ± 5.5	68.3 ± 5.6	14	—	2；3；4
Xu Chunyu, 2019 [14]	45/45	Xueshuantong injection + urokinase injection + low molecular weight heparin	Urokinase injection + low molecular weight heparin	25/20	24/21	59.12 ± 6.34	58.49 ± 6.28	14	Random number table	5；6；8
Chen Kui, 2018 [15]	34/34	Xueshuantong injection + urokinase injection + low molecular weight heparin	Urokinase injection + low molecular weight heparin	19/15	18/16	53.96 ± 18.95	54.14 ± 19.02	14	Admission sequence	1; 5；6；7；8
Pan Lei, 2018 [16]	63/63	Xueshuantong injection + rivaroxaban	Rivaroxaban	32/31	33/30	65.09 ± 4.12	65.24 ± 4.27	14	Random number table	1；7；9
Zhang Yanwen, 2018 [17]	25/25	Xueshuantong injection + low molecular weight heparin	Low molecular weight heparin	14/11	15/10	67.83 ± 7.24	67.19 ± 6.25	14	—	1；7；8；9
Zhou Bo, 2015 [18]	44/43	Xueshuantong injection + low molecular weight heparin + warfarin	Low molecular weight heparin + warfarin	30/14	29/14	64.3 ± 5.2	64.8 ± 5.5	10	—	1；8
Zhou Yuhu, 2015 [19]	39/39	Xueshuantong injection + rivaroxaban	Rivaroxaban	21/18	20/19	66.52 ± 3.63	66.61 ± 3.72	14	—	1；5；6；7；8；9
Zhang Jianping, 2012 [20]	28/28	Xueshuantong injection + urokinase injection + low molecular weight heparin + aspirin	Urokinase injection + low molecular weight heparin + aspirin	18/10	15/13	57.25 ± 13.64	55.31 ± 14.26	14	Random number table	2；3；4；9

*Note.* ① Clinical efficiency. ② Patency rate of femoral vein. ③ Patency rate of the popliteal vein. ④ Patency rate of the posterior tibial vein. ⑤ Circumference difference of upper knee limb. ⑥ Circumference difference of lower knee limb. ⑦ APTT. ⑧ D-D. ⑨ Incidence of adverse events.

## Data Availability

The data used to support the findings of this study are included within the article.

## Abbreviations

RCTs: Randomized clinical trials
CNKI: China national knowledge infrastructure
VIP: Chinese scientific journal database
CI: Confidence interval
MD: Mean difference
RR: Relative risk
DVT: Deep venous thrombosis
PRISMA: Preferred Reporting Items for Systematic Reviews and Meta-Analyses
SMD: Standardized mean difference
APTT: Activated partial thromboplastin time
D-D: The D-dimer
OR: Odds ratio
TSA: Trial Sequential Analysis
RIS: Required information size.
